# Differences in immune responses between CMV-seronegative and -seropositive patients with myocardial ischemia and reperfusion

**DOI:** 10.1002/iid3.49

**Published:** 2015-03-01

**Authors:** Evgeniya V Shmeleva, Stephen E Boag, Santosh Murali, Karim Bennaceur, Rajiv Das, Mohaned Egred, Ian Purcell, Richard Edwards, Stephen Todryk, Ioakim Spyridopoulos

**Affiliations:** 1 Institute of Genetic Medicine, Newcastle UniversityNewcastle upon Tyne, UK; 2 Department of Applied Sciences, Faculty of Health & Life Sciences, Northumbria UniversityNewcastle upon Tyne, UK; 3 Institute of Cellular Medicine, Newcastle UniversityNewcastle upon Tyne, UK; 4 Department of Cardiology, Freeman HospitalNewcastle upon Tyne, UK; 5 Institute of Ageing and Health, Newcastle UniversityNewcastle upon Tyne, UK

**Keywords:** CMV infection, cytokines, myocardial infarction, reperfusion injury, T cells

## Abstract

CMV infection is responsible for acceleration of immune senescence and linked to systemic pathologies, including cardiovascular diseases. In this study, we investigated differences in the immune response between CMV-seropositive and seronegative patients undergoing primary percutaneous coronary intervention (PPCI) for acute myocardial infarction (MI). Peripheral blood samples were taken at six different time points: pre-, 15, 30, 90 min, 24 h after PPCI and at 3 months after MI. Absolute counts of lymphocyte subpopulations, immune response to specific and nonspecific stimulation, serum cytokines and levels of CMV-IgG, cardiolipin-IgG, and anti-endothelial cell antibodies were assessed. CMV-seropositive patients with MI showed a twofold higher IFN-γ production to PHA-stimulation, up to 2.5-fold higher levels of IP-10 in serum and up to 30% lower serum levels of IL-16 compared to CMV-seronegative individuals. CMV-seropositive patients could be divided into two subgroups with high (IL-10Hi) and low (IL-10Lo) IL-10 serum levels during the acute stage of MI. The IL-10Hi CMV-seropositive subgroup showed an increased exit of late-differentiated T lymphocytes, NK and NKT-like cells from the circulation, which may potentially enhance cytotoxic damage in the ischemic myocardium. Finally, we did not observe an acceleration of autoimmunity by MI in CMV-seropositive individuals. The immune response during acute MI showed characteristic differences between CMV seronegative and seropositive patients, with a stronger pro-inflammatory response in seropositive patients. The effects of IP-10, IL-16, and IL-10 on characteristics of acute immune responses and formation of different immune profiles in CMV-seropositive individuals require further investigation.

## Introduction

Every year approximately 25,000 patients in the UK undergo reperfusion therapy for acute myocardial infarction, in most cases by primary percutaneous coronary intervention (PPCI), which involves opening of the blocked coronary artery followed by placing a stent 1. Major recent improvements in PPCI and associated therapies have lead to a reduction in acute mortality. However, morbidity and mortality following hospital discharge remains significant 1,2. There are two major reasons for this, the first of which relates to the size of the infarct, culminating in left ventricular dysfunction and eventually leading to heart failure. The other is the accelerated progression of atherosclerosis following myocardial infarction (MI), which could be explained by the pro-inflammatory state of such patients. While the immune system, and in particular lymphocytes, regulate inflammation, their role in myocardial infarction has not been investigated in detail.

Human cytomegalovirus (CMV) is a ubiquitous herpes virus that even after recovery from acute infection is never cleared from the human body. Chronic CMV infection leads to phenotypic changes in the immune system, most visible in the T-cell compartment, coinciding with clonal expansion and preferential accumulation of CD8^**+**^CD45RA^**+**^CD27^−^ cytotoxic T cells 3,4. Clinically, CMV has been linked to an increased incidence of coronary heart disease and an increased risk of cardiovascular death in people over the age of 65 years without an increase in other causes of mortality 5. We have shown previously a link between immunosenescence and coronary artery disease 6,7.

We have also recently published on the impact of the myocardial ischemia and reperfusion on absolute counts of T cells in peripheral blood: predominantly CD4^+^ and CD8^+^ late-differentiated T-cell subpopulations fell dramatically within the first hour after reperfusion, followed by a increase to prior PPCI levels at the 24-h time point 6,8.

We hypothesized that a higher proportion of the late-differentiated T-cell populations leave the blood circulation after reperfusion in MI in CMV-seropositive patients than in CMV-seronegative patients, which may have a negative effect on reperfusion injury in MI leading to a larger infarct size.

The aim of this study was to investigate differences in the immune response between CMV seropositive and seronegative patients undergoing reperfusion therapy for acute myocardial infarction.

## Material and Methods

### Patient population

A cohort of 52 patients with ST elevation myocardial infarction (STEMI) were prospectively identified and enrolled in the study at the time of admission. Inclusion criteria were chest pain of onset within 6 h with new ST segment elevation on electrocardiogram. Exclusion criteria were cardiogenic shock, previous MI, active infection or malignancy, chronic inflammatory conditions, patent arterial flow in the infarct related artery, and any exclusion to cardiac MRI. Written informed consent was obtained from all patients. Patient characteristics are displayed in supplemental Table S1. The study protocol was approved by the NHS HRA committee (REC 12/NE/0322).

### Blood sampling

Arterial blood was taken before reperfusion and at 15, 30, and 90 min following reperfusion. Venous blood samples were obtained at 24 h and after 3–6 months post-PPCI.

### Clinical tests

CMV and EBV serostatus as well as baseline values of general blood analysis, lipid panel, glucose, creatinine, and 12-h troponin levels were assessed at the Freeman Hospital Laboratory.

### Flow cytometry

All FACS analysis was performed using BD FACS Canto II flow cytometer with FACSDiva software (BD Biosciences, San Jose, CA, USA). A minimum of 10,000 events for a population of interest were recorded for each sample.

### Identification of lymphocyte subpopulations

Patients' blood was collected in EDTA tubes (BD Biosciences) at 6 time points: pre-, 15, 30, 90 min and 24 h post reperfusion as well as 3 months after the acute event. Whole blood samples were used for quantification of absolute counts and percentage distribution of lymphocyte subpopulations by multicolor flow cytometry. Absolute counts of granulocytes, monocytes, CD4^+^ and CD8^+^ T lymphocytes were measured using the BD MultiTEST cocktail (anti-CD3-FITC, CD8-PE, CD45-PerCP, CD4-APC) and BD Trucount tubes (BD Biosciences). NK and NKT-like cells as well as naïve (CCR7^+^CD45RA^+^), central memory (CCR7^+^CD45RA^−^), effector memory (CCR7^−^CD45RA^−^) and terminally differentiated effector memory (CCR7^−^CD45RA^+^) subpopulations of CD4^+^ and CD8^+^ T lymphocytes were identified with the following monoclonal antibodies: anti-CD3-FITC, CD4-V500, CD8-APC-H7, CD16-PE, CD27-APC, CCR7(CD197)-PE-Cy7 (BD Biosciences), CD45RA-Pacific Blue (Invitrogen) and CD56-PerCP-eFluor710 (eBioscience, San Diego, CA, USA). Following incubation with these antibodies, samples were lysed, washed and immediately analyzed on a flow cytometer.

### Quantification of regulatory T cells

Regulatory T cells (Tregs) were measured in EDTA whole blood samples collected at 3 time points only: pre-reperfusion and 90 min and 24 h post reperfusion. Firstly, for surface staining, blood samples were incubated with anti-CD3-PerCP, CD4-V500, CD25-APC (BD Biosciences) monoclonal antibodies, lysed by BD Pharm Lyse (BD Biosciences) and washed twice with PBS containing 5% fetal calf serum (5% FBS/PBS). After fixation and permeabilization (BD Pharmigen Human FoxP3 Buffer Set, BD Biosciences), cells were incubated with an anti-FoxP3-PE monoclonal antibody (BD Biosciences) for intracellular staining. Finally, samples were washed and analyzed on a flow cytometer. PE Mouse IgG1, κ Isotype (BD Biosciences) was used to exclude nonspecific binding.

### Measurement of CMV-specific CD8^+^T cells

HLA typing of CMV-seropositive patients was performed by NHS Blood and Transplant Newcastle. CMV-specific CD8^+^ T cells were identified in PBMCs isolated from pre-PPCI, 90 min, 24 h and 3-month blood samples as published previously 6. In brief, cryopreserved PBMCs were quickly thawed and washed with 5% FBS/PBS before cell viability was determined using a Vi-CELL Cell Viability Analyzer (Beckman Coulter). 1 × 10^6^ cells then were incubated with HLA-matched MHC Dextramer-PE (Dextramer CMV Kit, Immudex) for 10 min, followed by 20 min incubation with anti-CD3-FITC, CD27-APC, CD8-APC-H7, CD45RA-PacificBlue (BD Biosciences). Finally, cells were washed twice with 5% FBS/PBS and assessed by FACS. PE-Negative control (Dextramer CMV Kit, Immudex) was used to exclude nonspecific binding.

### ELISPOT assay

Cryopreserved PBMC were quickly thawed, washed and resuspended in RPMI 1640 medium (Sigma, Dorset, UK) supplemented with penicillin (100 U/mL), streptomycin (100 mg/mL), L-glutamine (2 mM) (Invitrogen, Paisley, UK) and 10% heat-inactivated FBS (Biosera, Uckfield, UK). PBMC were set up for ex-vivo ELISPOT at 8 × 10^6^/mL (50 mL/well of capture antibody-coated Millipore plate) for 20 h incubation, as optimized and described previously 6,9. ELISPOT kits were purchased from Mabtech (Nacka, Sweden) and manufacturer's instructions followed for plate development, with modifications as previously described 9. The CMV and EBV viral antigenic stimuli used, were pools of known CD8 epitope peptides (10 µg/mL) for CD8 responses 10 and pools of known CD4 epitope peptides (10 µg/mL) plus viral lysates for CD4 responses. Medium-only and phaetohaemagglutinin (PHA, 5 µg/mL) controls were used in all assays. Measurement of polyclonal secretion of IL-5, IL-17 and IL-2 was carried out using PHA as the stimulus, and ELISPOT reagents from Mabtech (IL-5), eBioscience (IL-17), or R&D Sytems (IL-2), again following manufacturer's instructions. Results are expressed as spot-forming cells/10^6^ PBMC (net antigen-stimulated spots minus medium).

### Cytokine measurement

Thirty one cytokines were assessed in serum samples (pre-, 15, 30, 90 min, 24 h and 3-month time points) using human the IL-18 kit and human cytokine 30-Plex Kit, V-PLEX, Meso Scale Discovery (containing IFN-γ, IL-1β, IL-2, IL-4, IL-6, IL-8, IL-10, IL-12p70, IL-13, TNFα, GM-CSF, IL-1α, IL-5, IL-7, IL-12/IL-23p40, IL-15, IL-16, IL-17A, TNF-β, VEGF, Eotaxin, MIP-1β, Eotaxin-3, TARC, IP-10, MIP-1α, MCP-1, MDC, MCP-4) on a SECTOR Imager instrument (Meso Scale Discovery, Rockville, MD, USA) according to manufacturers' protocols.

### ELISA assays

ELISA kits were used to quantify CMV IgG-specific antibodies in CMV-seropositive patients (Cat. Number: 40-052-115031, GenWay Biotech) and detect human anti-endothelial cell antibody, AECA (Cat. Number: CSB-E08691h, CUSABIO) as well as anti-cardiolipin IgG antibody (Cat. Number: 40-101-325062, GenWay Biotech) according to the manufacturers' protocols.

### Statistical analysis

SPSS version 21 and GraphPad Prism version 6 were used for statistical analysis. For comparisons between two groups non-parametric Mann–Whitney *U*-test and paired Wilcoxon test were used, and for analysis of more than two groups two-way repeat measurements (RM) ANOVA tests. *P* values <0.05 were considered significant. Correlations were analyzed by Spearman's correlation analysis. Corresponding tests are indicated in the text. Data are presented as median with low and upper quartiles.

## Results

### Baseline characteristics of patient population

All 52 patients with acute myocardial infarction (STEMI) underwent reopening of their occluded coronary artery by PPCI, leading to immediate reperfusion. As expected, CMV-seropositive patients were slightly older than CMV-negative patients (62 vs. 56 years, *P* = 0.033, supplemental Table S1). Clinical tests included a full blood analysis, lipids, creatinine, glucose, and troponin levels (Supplemental Table S1). Infarct size, estimated by peak troponin levels, did not differ between the two study groups (4800 vs. 5098, *P* = 0.93).

### Lymphocyte subpopulations in CMV-seropositive and seronegative patients

**Figure 1:**
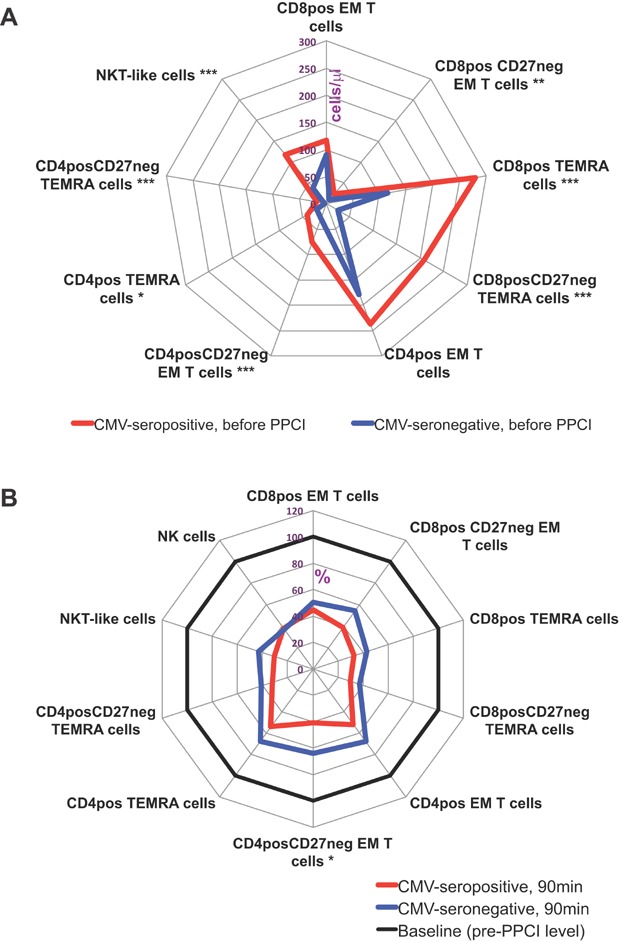
Lymphocyte subpopulations in CMV-seropositive and seronegative patients. Absolute counts of different lymphocyte subpopulations in CMV-seropositive and seronegative patients before PPCI (A). Cell counts of different lymphocyte subpopulations at 90 min after PPCI relative to their pre-PPCI levels (% from baseline) (B). Data are presented as medians. *P values by Mann–Whitney test for CMV-seropositive and seronegative patients; *P < 0.005; **P < 0.01; ***P < 0.001. EM, effector memory; TEMRA, CD45RA^+^ effector memory T cells.

Our results demonstrated increased absolute counts of late-differentiated effector memory (EM) and CD45RA^+^ effector memory (TEMRA) CD4^+^ and CD8^+^ T lymphocytes (CD4/8^+^CD45RA^−/+^CCR7^−^CD27^−^ T-subsets), as well as NKT-like cells (CD3^+^CD56^+^) in CMV-seropositive individuals, in keeping with previously published findings (Fig. 1A) 11,12. This pattern persisted across all time points of acute MI (Supplemental Table S2). CMV-seropositive and seronegative patients showed a significant cell loss of CD4^+^ and CD8^+^ CD45RA^+/−^CCR7^−^CD27^−^ T-subpopulations as well as NK and NKT-like cells during 90 min after reperfusion, followed by a gradual increase to prior-PPCI levels at the 24-h time point. This trend was the same for both CMV-seropositive and seronegative patients (Supplemental Table S2). Furthermore, the subpopulations had similar percentages of cell drops in both groups of patients (on average 40–60%; Fig. 1B). At 3 months, the described cell types were fully recovered.

**Figure 2:**
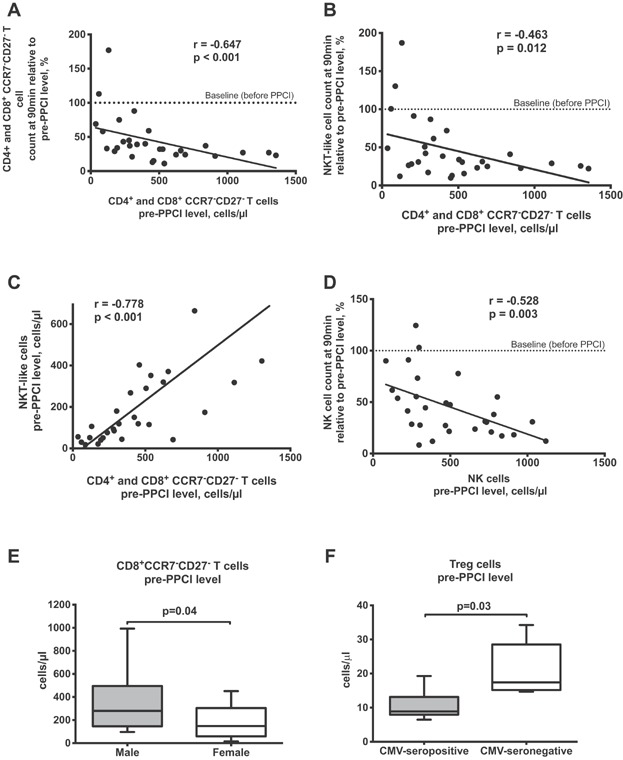
T lymphocytes, NK and NKT-like cells in CMV-seropositive patients. Spearman correlation analysis for absolute count of highly differentiated T cells and their 90 min results relative to its pre-PPCI level (A), and 90 min results of NKT-like cells relative to its pre-PPCI level (B) in CMV-seropositive patients. Spearman correlation analysis between absolute counts of CCR7^−^CD27^−^ T cells and NKT-like cells before PPCI in CMV-seropositive patients (C). Spearman correlation analysis for absolute count of NK cells and their 90 min results relative to its pre-PPCI level in CMV-seropositive patients (D). Dashed lines (at 100-tick) indicate a baseline representing the level of corresponding parameter before PPCI. Absolute count of CD8^+^CCR7^−^CD27^−^ T cells in male and female CMV-seropositive patients (E). Absolute count of Treg cells in CMV-seropositive and seronegative patients (F). Boxplots represent median, lower/upper quartiles and min/max values. *P* value by Mann-Whitney test.

Among CMV-seropositive patients, there was a wide variation regarding the total numbers of CD4^+^ and CD8^+^ effector memory/TEMRA T cells. Correlation analysis revealed an association between the number of late differentiated T cells and their drop following reperfusion (Fig. 2A), as well as that of NKT-like cells (Fig. 2B). Moreover, absolute counts of NKT-like cells positively correlated with the level of CCR7^−^CD27^−^ T cells (Fig. 2C). The pre-PPCI number of NK cells also correlated with their drop (Fig. 2D).

CMV-seropositive men showed higher levels of CD8^+^CCR7^−^CD27^−^ T cells than CMV-seropositive women (Fig. 2E). Furthermore, there was a positive correlation between the peak troponin level, reflecting infarct size, and the magnitude of drop in CD4^+^CCR7^−^ T lymphocytes for CMV-seropositive men (Spearman correlation; *r* = −0.491, *P* = 0.039) (data not presented).

Regulatory T cells (Treg; CD3^+^CD4^+^CD25^high^FoxP3^+^) were measured before as well as 90 min and 24 h after reperfusion. There was a significant difference in the absolute count of Treg cells only before reperfusion, with CMV-seropositive patients showing lower Treg cell levels than CMV-negative individuals (Fig. 2F, Supplemental Table S2).

### CD8^+^ CMV-specific T-cell dynamics

**Figure 3:**
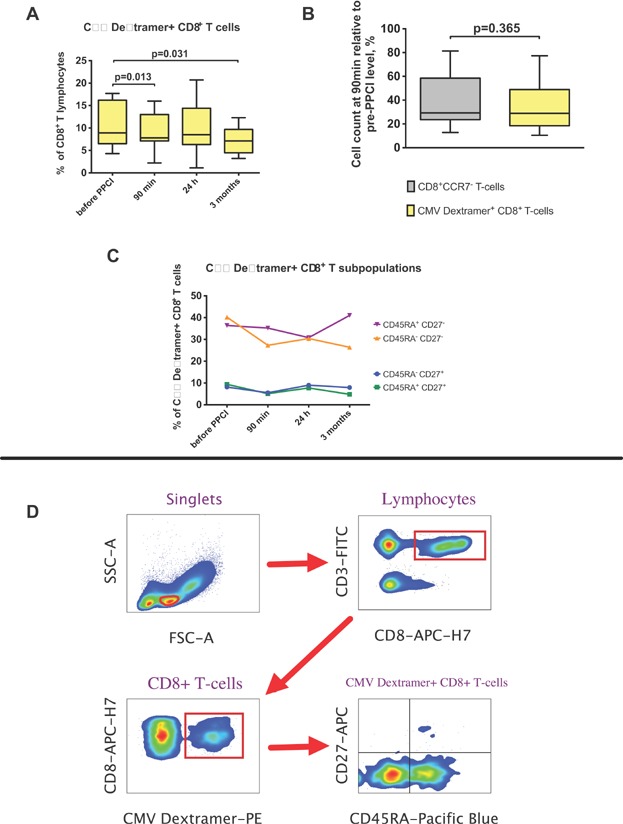
Dynamic of CMV-specific CD8^+^ T cells. Percentages of CMV dextramer^+^ CD8^+^ T lymphocytes at different time points (A). 90 min levels of CD8^+^CCR7^−^ T cells and CMV-specific CD8^+^ T lymphocytes relative to their pre-PPCI level (B). Boxplots represent median, lower/upper quartiles and min/max values. *P* values by paired Wilcoxon test. Dynamic of percentages of CD45^+/−^CD27^+/−^ subpopulations from CMV dextramer^+^ CD8^+^ T cell at different time points (Medians represented) (C). (D) Example of gating strategy for FACS analysis of CMV dextramer^+^ CD8^+^ T-lymphocyte subpopulations.

We next analyzed the dynamics of CMV-specific CD8^+^T cells (CD3^+^CD8^+^CMV dextramer^+^) following reperfusion. The percentage of CMV dextramer^+^ CD8^+^T cells declined at 90 min and returned to pre-PPCI levels at the 24-h time point (Fig. 3A). Interestingly, the percentage of CMV-specific cells among CD8^+^ T lymphocytes at 3 months was significantly lower than in acute MI before PPCI. The percentage drops of CD8^+^ effector memory/TEMRA T cells and CMV-specific CD8^+^ T cells were similar (Fig. 3B).

Subpopulation analysis revealed that CD27^−^ cells made up 80% of CD8^+^CMV dextramer^+^ T cells in MI (Fig. 3C and D). The composition of CMV-specific cells did not show significant changes during the acute stage of MI.

### ELISPOT results

**Figure 4:**
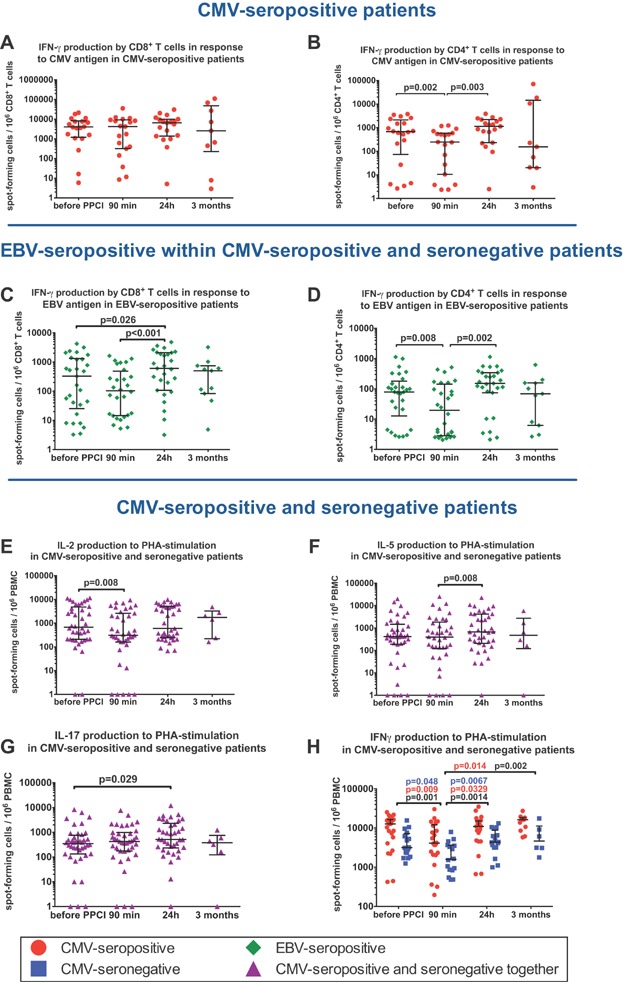
ELISPOT results at different time points. CD8^+^ (A) and CD4^+^ (B) T-cell response to 24 h stimulation with CMV-antigen in CMV-seropositive patients. CD8^+^ (C) and CD4^+^ (D) T cell response to 24 h stimulation with EBV-antigen in EBV-seropositive patients. Production of IL-2 (E), IL-5 (F), IL-17 (G) and IFNγ (H) as responses to 24 h PHA-stimulation in CMV-seropositive and seronegative patients. In Figure 3H, *P* values are provided separately for CMV-seropositive individuals (red), CMV-seronegative individuals (blue) and for CMV-seropositive and seronegative individuals together (black). Horizontal bars represent medians with lower and upper quartiles. *P* values by paired Wilcoxon test for different time points.

To characterize the antigen-specific response functionally, we measured IFN-γ production by CD4^+^ and CD8^+^ T lymphocytes using ELISPOT. While the CMV-specific CD8^+^ T-cell response remained unchanged across all time points (Fig. 4A), we saw a transient drop in the CD4^+^ T-cell immune response to CMV-peptide stimulation at 90 min after PPCI (Fig. 4B).

To investigate the immune response against other herpes viruses we stimulated PBMCs of EBV-seropositive patients with CD8 or CD4 epitope EBV peptides. CMV-seropositive and seronegative individuals did not differ in levels of CD4^+^ and CD8^+^ T-cell EBV-specific responses (data not shown). The reaction to EBV-peptides declined at 90 min after PPCI in CMV-seropositive and seronegative patients, regardless of CMV serostatus (Fig. 4C and D). As expected, the CMV-specific response was much stronger than the response to EBV-stimulation, especially for CD8^+^ T lymphocytes. Importantly, IFN-γ secretion by CD8^+^ T cells was approximately 10-fold higher than by CD4^+^T lymphocytes.

To investigate differences in nonspecific T-cell responses, we measured PHA-induced production of IL-2, IL-5, IL-17, and IFN-γ (Fig. 4E–G). There were no differences in IL-2, IL-5, and IL-17 secretion between CMV-seropositive and seronegative individuals. However, CMV-seropositive patients had a two to threefold higher PHA-induced IFN-γ secretion (*P* = 0.0002; two-way RM ANOVA) across all time points than CMV-seronegative individuals. Furthermore, there was a significant drop of IFN-γ secretion in both patient groups at 90 min after reperfusion, which returned to baseline levels after 24 h (Fig. 4H).

### Serum cytokine levels in CMV-seropositive and seronegative patients

**Figure 5:**
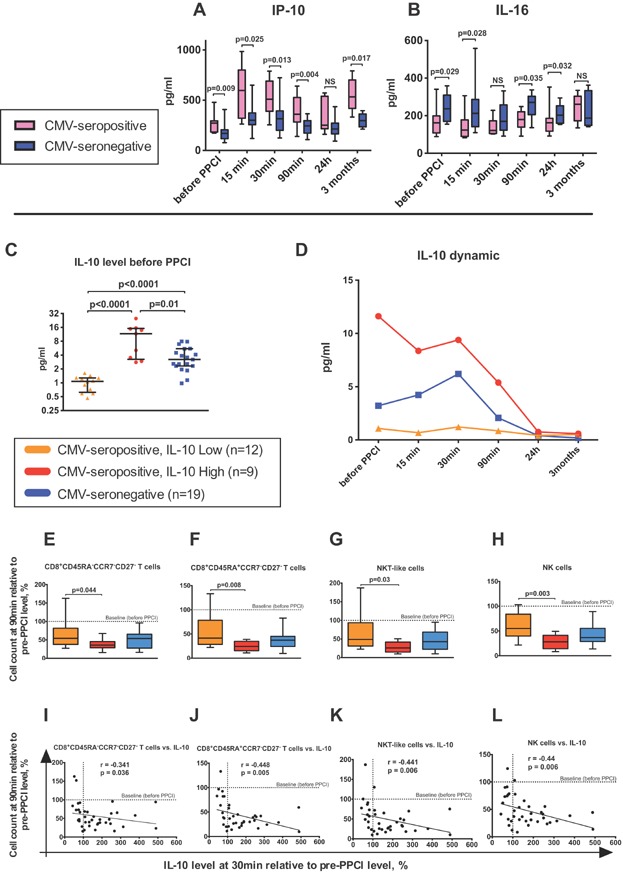
Serum levels of IP-10, IL-16 and IL-10 in CMV-seropositive and seronegative individuals. Serum levels of IP-10 (A) and IL-16 (B) in CMV-seropositive and seronegative patients at different time points. Boxplots represent median, lower/upper quartiles and min/max values. *P* values by Mann-Whitney. NS, not significant. IL-10 serum level before PPCI in IL-10Lo and IL-10Hi subgroups of CMV-seropositive patients as well as CMV-seronegative individuals (C). Horizontal bars represent medians with lower and upper quartiles. *P* values by Mann–Whitney. Dynamics of IL-10 levels in CMV-seronegative and IL-10Lo, IL-10Hi subgroups of CMV-seropositive patients (Median is depicted for each time point) (D). Cell counts at the 90 min time point relative to their pre-PCCI levels: CD8+CD27- effector memory (E), CD8^+^TEMRA CD27^−^ T cells (F), NKT-like cells (G) and NK-cells (H) in CMV-seronegative and IL-10Hi, IL-10Lo subgroups of CMV-seropositive patients. Boxplots represent median, lower/upper quartiles and min/max values. *P* values by Mann–Whitney. Spearman correlation analysis between IL-10 changes (Δ pre-30 min after PPCI) and changes (Δ pre-90 min after PPCI) of CD8+CD27- effector memory T cells (**I**), CD8^+^TEMRA CD27^−^ T cells (J), NKT-like cells (K) and NK-cells (L) for CMV-seronegative and IL-10Hi, IL-10Lo subgroups of CMV-seropositive patients. Dashed lines (at 100-tick) indicate a baseline representing the level of corresponding parameter before PPCI.

Among 31 detected serum cytokines, CMV-seropositive and seronegative patients differed only in pre- and post-reperfusion levels of IL-16 (*P* = 0.0003; two-way ANOVA) and IP-10 (*P* < 0.0001; two-way ANOVA) in acute MI. CMV-seropositive patients had higher levels of IP-10 and lower level of IL-16 (Table1, Fig. 5A and B). Also, the dynamics of IP-10 changes were opposite to the cell drops at the measured time points. More pronounced changes were registered within the first 15 min after reperfusion, when IP-10 increased up to twofold. However, there were no significant correlations between cell counts and IP-10 levels (data not presented).

**Table 1:** Serum levels of IP-10 and IL-16 in CMV-seropositive and seronegative patients at different time points

CMV status	Time points	Median (25% quartile; 75% quartile), pg/mL	p1	p2	p3	p4	p5
IL-16							
Positive	Acute MI, *n* = 11						
	Before PPCI	162 (108; 199)					
	After PPCI						
	15 min	137 (92; 216)	0.322				
	30 min	125 (106; 210)	0.770	0.922			
	90 min	191 (149; 228)	0.123	0.375	0.160		
	24 h	162 (117; 186)	0.520	0.846	0.322	0.083	
	3 months after acute MI, *n* = 5	261 (173; 304)	0.145	0.129	0.055	0.267	0.052
Negative	Acute MI, *n* = 14						
	Before PPCI	237 (170; 309)					
	After PPCI						
	15 min	213 (141; 286)	0.424				
	30 min	170 (123; 257)	0.007^*^	0.339			
	90 min	271 (206; 303)	0.636	0.240	0.011^*^		
	24 h	203 (160; 253)	0.204	>0.999	0.339	0.151	
	3 months after acute MI, *n* = 6	187 (148; 333)	0.518	0.918	0.493	0.606	0.849
IP-10							
Positive	Acute MI, *n* = 11						
	Before PPCI	272 (183; 294)					
	After PPCI						
	15 min	608 (338; 964)	0.002^*^				
	30 min	549 (416; 726)	0.004^*^	0.695			
	90 min	418 (284; 602)	0.005^*^	0.01^*^	0.02^*^		
	24 h	250 (215; 539)	0.054	0.002^*^	0.006^*^	0.102	
	3 months after acute MI, *n* = 5	533 (418; 701)	0.002^*^	0.554	>0.999	0.180	0.052
Negative	Acute MI, *n* = 14						
	Before PPCI	169 (116; 205)					
	After PPCI						
	15 min	301 (256; 373)	0.002^*^				
	30 min	315 (200; 394)	<0.001^*^	0.519			
	90 min	244 (172; 296)	0.013^*^	0.003^*^	0.005^*^		
	24 h	213 (164; 271)	0.034^*^	0.084	0.034^*^	0.622	
	3 months after acute MI, *n* = 6	297 (234; 362)	0.005^*^	0.647	0.863	0.152	0.053

*P* values for different time points by paired Wilcoxon test. p1: *P* values for corresponding time point and parameter before PPCI; p2: *P* values for corresponding time point and parameter at 15min after reperfusion; p3: *P* values for corresponding time point and parameter at 30min after reperfusion; p4: *P* values for corresponding time point and parameter at 90 min after reperfusion; p5: *P* values for corresponding time point and parameter at 24 h after reperfusion. Significant values in bold.

The serum concentration of IL-16 fluctuated slightly at the acute stage of MI (Table1). Furthermore, there was an inverse correlation between the magnitude of the CD4^+^CCR7^+/−^ T-lymphocyte drop and the dynamics of IL-16 secretion: the more IL-16 increased in the period up to 90 min after PPCI (Δ pre-90 min after PPCI), the more CD4^+^ T lymphocytes declined at the same time interval (Spearman correlation; *r* = −0.523, *P* = 0.009) (Supplemental Fig. S1). Also, the high level of IL-16 at the 90 min time point was associated with a more pronounced decrease of IFN-γ response to PHA-stimulation (Δ pre-90min after PPCI) (Spearman correlation; *r* = −0.521, *P* = 0.027).

Serum samples taken at 3 months contained higher concentrations of IP-10 (533 vs. 297 pg/mL; *P* = 0.017, Mann–Whitney), IL-17A (3.29 vs. 1.04 pg/mL; *P* = 0.008, Mann–Whitney) and IL-10 (0.51 vs. 0.19 pg/mL; *P* = 0.008, Mann–Whitney) in CMV-seropositive compared to seronegative patients.

### IL-10Hi and IL-10Lo subgroups of CMV-seropositive patients

Serum levels of IL-10 were measured in 40 patients. As indicated above, CMV-seropositive and seronegative patients had different levels of IL-10 only at the 3-month time point. Nonetheless, since the CMV-seropositive group showed heterogeneous results for serum levels of IL-10, we compared patients with high (IL-10Hi; *n* = 9) and low (IL-10Lo; *n* = 12) levels of IL-10 (Fig. 5C–L). These two CMV-seropositive subgroups had significantly different serum concentrations of IL-10 (*P* = 0.002; two-way RM ANOVA) in the acute stage of MI (Fig. 5C). Also, IL-10Hi and IL-10Lo subgroups could be distinguished from CMV-seronegative patients (*P* = 0.01 and *P* = 0.004, respectively; two-way RM ANOVA). As shown in Figure 5D, IL-10 concentration in the IL-10Lo CMV-seropositive subgroup remained at a constant low, while the other patients had high IL-10 levels before as well as 15, 30, and 90 min after reperfusion. However, IL-10Hi CMV-seropositive patients had almost a threefold higher magnitude of IL-10 than CMV-seronegative study participants. The described differences were observed only during the acute stage of MI, yet lL-10 levels were equal at 3 months in both subgroups of CMV-seropositive subjects and were higher than in CMV-seronegative individuals (stated above) (Supplemental Fig. S2).

Interestingly, glucose levels were higher in the IL-10Hi than in the IL-10Lo subgroup (9.2 vs. 6.2; *P* = 0.018, Mann–Whitney). Furthermore, the cell decrease described above was more pronounced for IL-10Hi CMV-seropositive patients. Figure 5E–H illustrates significant differences in the magnitude of cell drops for CD8^+^CD45RA^+/−^CCR7^−^CD27^−^ T lymphocytes, NK and NKT-like cells between the subgroups. Also, the change in serum IL-10 concentration after PPCI correlates with the drops of these lymphocyte subpopulations (Fig. 5I–L).

In addition, IL-10Hi patients had a very sharp increase of IP-10 levels in serum after 15 min of reperfusion, in contrast to the IL-10Lo subgroup (828 (IL-10Hi) versus 484 (IL-10Lo); *P* = 0.044, Mann–Whitney).

### Autoimmunity and MI

To evaluate a potential impact of MI on autoimmune drive, the presence of anti-endothelial cell antibodies (AECA) and cardiolipin autoantibodies were measured in 24 h and 3-month samples using ELISA assays. AECA were found in 3 CMV-seropositive and 3 CMV-seronegative patients. One of these CMV-seronegative individuals had AECA at the 3-month time point only, but not during the acute stage of MI. Anti-cardiolipin antibodies were detected in 6 CMV-seropositive patients and 7 CMV-seronegative patients at both time points.

In addition, we measured CMV IgG antibodies in serum samples taken at the 24 h and 3-month time points. No increase of CMV specific IgG titer was registered at 3 months after MI.

## Discussion

We have shown previously that reopening of an occluded coronary artery with PPCI during acute MI leads to an acute depletion of T cells from peripheral blood 6. This was associated with lymphocyte exit from the circulating bloodstream. In this study, we demonstrate that CMV-seropositive and seronegative patients show different dynamics in nonspecific IFN-γ production, as well as IP-10 and IL-16 expression in the serum during acute MI. In addition, CMV-seropositive patients with high IL-10 levels demonstrated an increased drop of cytotoxic lymphocyte populations during reperfusion, which may suggest a worse clinical outcome, given the previously published association between low lymphocyte counts and increased mortality in MI 13.

### Depletion of cytotoxic lymphocyte populations during acute MI

Since CMV-seropositive individuals had higher baseline numbers of late-differentiated T lymphocytes and NKT-like cells, the absolute count of “missing” cells after reperfusion was much larger in CMV-seropositive than in CMV-seronegative patients. Furthermore, pre-PPCI absolute counts of CD45RA^+/−^CCR7^−^CD27^−^ T lymphocytes, NK and NKT-like cells negatively correlated with percentages of their drop. We speculate that these cells may be lost from the circulation in part through sequestration into inflamed tissues, including reperfused myocardium. Given that these lymphocyte populations are known to have cytotoxic activity 14,15, we hypothesized correlations between prominent cell loss and severity of the clinical picture and infarct size. However, there was only one significant correlation, which demonstrated the relationship between CD4^+^CCR7^−^ T-cell decline intensity and troponin level reflecting infarct size, but only for CMV-seropositive males.

The depletion of CMV-specific CD8^+^ T lymphocytes in blood from CMV-seropositive patients resembled the drop in late-differentiated T cells. This can be explained by the fact that approximately 80% of CMV dextramer^+^CD8^+^ T cells showed a CD45^+/−^CD27^−^ phenotype (late-differentiated CMV-specific CD8^+^ T lymphocytes). However, the balance of CD45^+/−^CD27^+^ and CD45^+/−^CD27^−^ CMV dextramer^+^ T-subpopulations remained constant within the acute stage of MI suggesting that the migratory ability of these subsets was equal.

### Antigen-specific and PHA-related response in CMV-seropositive and seronegative individuals

The ELISPOT analysis revealed a decrease in EBV-induced IFN-γ secretion by CD4^+^ and CD8^+^ T cells and CMV-induced IFN-γ secretion by CD4^+^ T lymphocytes at 90 min post reperfusion, while CD8^+^ T-cell IFN-γ production to CMV-antigen remained stable during all time points. Consequently, we concluded that the decrease in IFN-γ secretion was a result of cell anergy rather than a drop in number of IFN-γ-producing cells in the PBMC samples. A similar decline of cytokine secretion was observed when PBMCs were incubated with a non-specific stimulator (PHA). A decrease in cytokine production was observed only for IFN-γ and IL-2, but not for IL-5 and IL-17. T-cell anergy is an important mechanism of immune tolerance, which preserves immune homeostasis and prevents autoimmunity. T-cell anergy leads to decreased secretion of IL-2 and IFN-γ, reduced response to antigens and inhibition of proliferation as well as effector function 16,17. Recently we have published that upregulation of PD1 (an inhibitory receptor) expression on T lymphocytes leads to functional anergy and high susceptibility to apoptosis of CMV-specific cells in STEMI patients undergoing PPCI 6. In the current study, anergy was shown in EBV-specific responses of CD8^+^ and CD4^+^ T cells and CMV-specific response of CD4^+^ T cells at the 90 min time point. However, CD8^+^ CMV-specific T lymphocytes retained their full effector function for longer, suggesting a lack of self-termination mechanisms, which may lead to escalation of inflammation.

As for increased PHA-mediated IFN-γ secretion in CMV-seropositive subjects, this fact could be a result of an enhanced proportion of late-differentiated T lymphocytes and NKT-like cells secreting a high amount of IFN-γ 18.

### Low IL-16 level in serum of CMV-seropositive patients with acute MI

We describe for the first time a decrease of IL-16 levels in CMV-seropositive patients in the acute stage of MI. IL-16 is produced by CD8^+^ and CD4^+^ T lymphocytes, dendritic cells, eosinophils, mast cells, monocytes, fibroblasts, epithelial, and neural cells 19. IL-16 is widely known as a chemoattractant capable of inducing the migration of CD4^+^ T lymphocytes and other cells which express CD4: eosinophils, monocytes, dendritic cells 19,20. Our results also showed a correlation between the CD4^+^ T-cell drop and serum levels of IL-16, which may play a role in the capability of immune defense during pathogen invasion or termination of excessive inflammation via Treg attraction. Interestingly, McFadden and co-authors provided proof of preferential recruitment and expansion of Treg lymphocytes in inflammatory areas mediated by IL-16 21. In our study, CMV-seropositive patients had a lower absolute count of Treg cells than CMV-seronegative individuals in the acute phase of MI.

IL-16 not only has a chemoattractant function, but also has the ability to influence TCR-mediated activation, as it is a ligand for the CD4 molecule 22. It has been shown that IL-16 inhibits the mixed lymphocyte reaction 22 and has an immunosuppressive effect on Th2 response 23. In addition, administration of rIL-16 decreases IFN-γ, IL-1β, and TNF levels in engrafted human inflamed rheumatoid synovium 24. We also showed decreasing IFN-γ production in the ELISPOT assay when the IL-16 serum level was high, which might be explained by the fact that IL-16 links to the CD4 receptor. Nonetheless, a wide range of immunostimulatory/pro-inflammatory IL-16 effects have been described as well 19,25. Cruikshank and co-authors suggested a hypothesis where IL-16-mediated recruited cells would retain the ability to respond to cytokine, but not to antigen-specific activation 19. Acute MI causes aseptic inflammation 26; therefore downregulation of antigen-specific cells is required. Due to the fact that late-differentiated memory CD4^+^ T cells very often showed crossreactivity 27, alloreactivity 28 and autoreactivity 29–31, the lack of IL-16 and Treg lymphocytes may lead to enhanced inflammation and eventually promotion of autoimmunity.

### High IP-10 serum level in CMV-seropositive individuals

IP-10 (C-X-C motif ligand 10, CXCL10) is an IFN-γ-inducible chemokine secreted by numerous cell types including monocytes, neutrophils, endothelial cells, fibroblasts, keratinocytes, T lymphocytes, NK, and NKT-like cells 32. It acts though its receptor CXCR3 and leads to recruitment of T lymphocytes, NK cells, eosinophils, and monocytes 33. In our study, the more pronounced drop of late-differentiated T lymphocytes and NK cells might be a consequence of the fact that the chemokine receptor CXCR3 is predominantly expressed on activated memory T lymphocytes and NK cells 34. The increased IP-10 level in serum of CMV-seropositive patients in our study was not surprising, owing to the fact that the IFN-γ/IP-10 loop is known to contribute in other viral infections as well as autoimmune diseases 32,35–37. However, our results did not show correlations between IFN-γ and IP-10 levels, nor an increased level of IFN-γ per se in serum of CMV-seropositive individuals.

### Heterogeneity of CMV-seropositive patients and IL-10 serum levels

Secreted by dendritic cells, macrophages, T- and B-lymphocytes 38, IL-10 is widely considered an immunosuppressant. However, a large number of original articles and reviews are dedicated to the dual function of IL-10 39,40. Numerous papers demonstrate antitumor activity of IL-10 due to its effect on NK cells and cytotoxic T lymphocytes 41,42. IL-10 downregulates mainly CD4^+^ T cells, yet it promotes the cytotoxic ability of CD8^+^ T lymphocytes and especially NK cells and enhances IFN-γ production by these cells 42–44. Furthermore, it has been published that the immunosuppressive function of IL-10 is related mainly to naïve T cells via inhibition of the costimulatory receptor CD28 45. Mocellin and coauthors concluded that IL-10 is capable of suppressing naïve cell priming by dendritic cells, which leads to immune tolerance and downregulation of autoaggression, whereas it stimulates activity of antigen-experienced cells 46.

Importantly, patients from the IL-10Lo subgroup had much smaller drops of NK cells, CD8^+^ effector memory and TEMRA T lymphocytes, as well as NKT-like cells, than the IL-10Hi patients. Our results also showed that the IP-10 level in the IL-10Hi patients increased much faster after reperfusion than in the IL-10Lo subgroup. Several papers also support the concept of upregulation of cell migration-related genes by IL-10 41,43,47. In other words, the possibility of cytotoxic damage of ischemic myocardium was lower in IL-10Lo patients, which is certainly beneficial for patients with STEMI. Supporting this conclusion, Derhovanessian and coworkers showed that IL-10 production in response to antigen stimulation correlated with worse survival in CMV-seropositive elderly individuals 11. It is important to note that the two subgroups of CMV-seropositive patients had different levels of IL-10 only at the acute stage of MI, but not at the 3-month time point.

In this study, we performed a comprehensive analysis of differences in characteristics of immune reactions in CMV-seropositive and seronegative individuals at the acute stage of MI, which can serve as a good in vivo model for investigation of acute reactive inflammation in the human organism. We have shown that IL-10, IL-16, and IP-10 play a significant role in the determination of various immune profiles of CMV-seropositive and seronegative patients at the time of acute MI. With CMV-seropositivity, high levels of IL-10 and IP-10 in serum, as well as low concentrations of IL-16 may be deleterious for patients with STEMI. Thus, the influence of CMV infection on IL-16 and IL-10 dynamics in acute inflammation merits further investigation.

## Limitations

The described percentages of CD8^+^ CMV-specific T cells and their subpopulations were based on a restricted number of HLA types: A*0101, A*0201, A*0301, A*2402, B*0702, B*0801, B*3501.

## Supporting Information

**Figure S1.** Relation between the dynamic of IL-16 and CD4+ T-cell drop. **Figure S2.** IL-10 levels at 3 months after MI in CMV-seropositive and seronegative patients.

**Table S1.** Baseline characteristics of CMV-seropositive and seronegative groups of patients. **Table S2.** Absolute counts of lymphocyte populations in CMV-seropositive and seronegative patients at different time points.
