# Supplementary figures and images for: Differences in immune responses between CMV-seronegative and -seropositive patients with myocardial ischemia and reperfusion
Source: Immun Inflamm Dis. 2015 Mar 1;3(2):56–70. doi: 10.1002/iid3.49 (PMC4444149; doi:10.1002/iid3.49)

Supplemental Figure 1

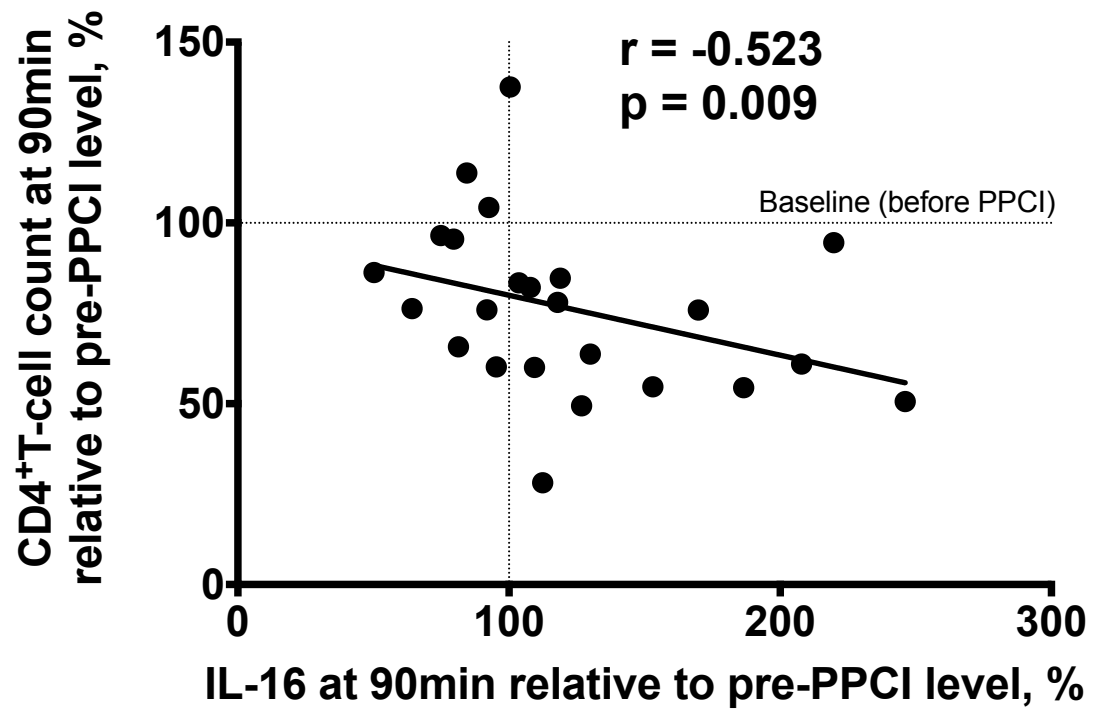

Supplemental Figure 2

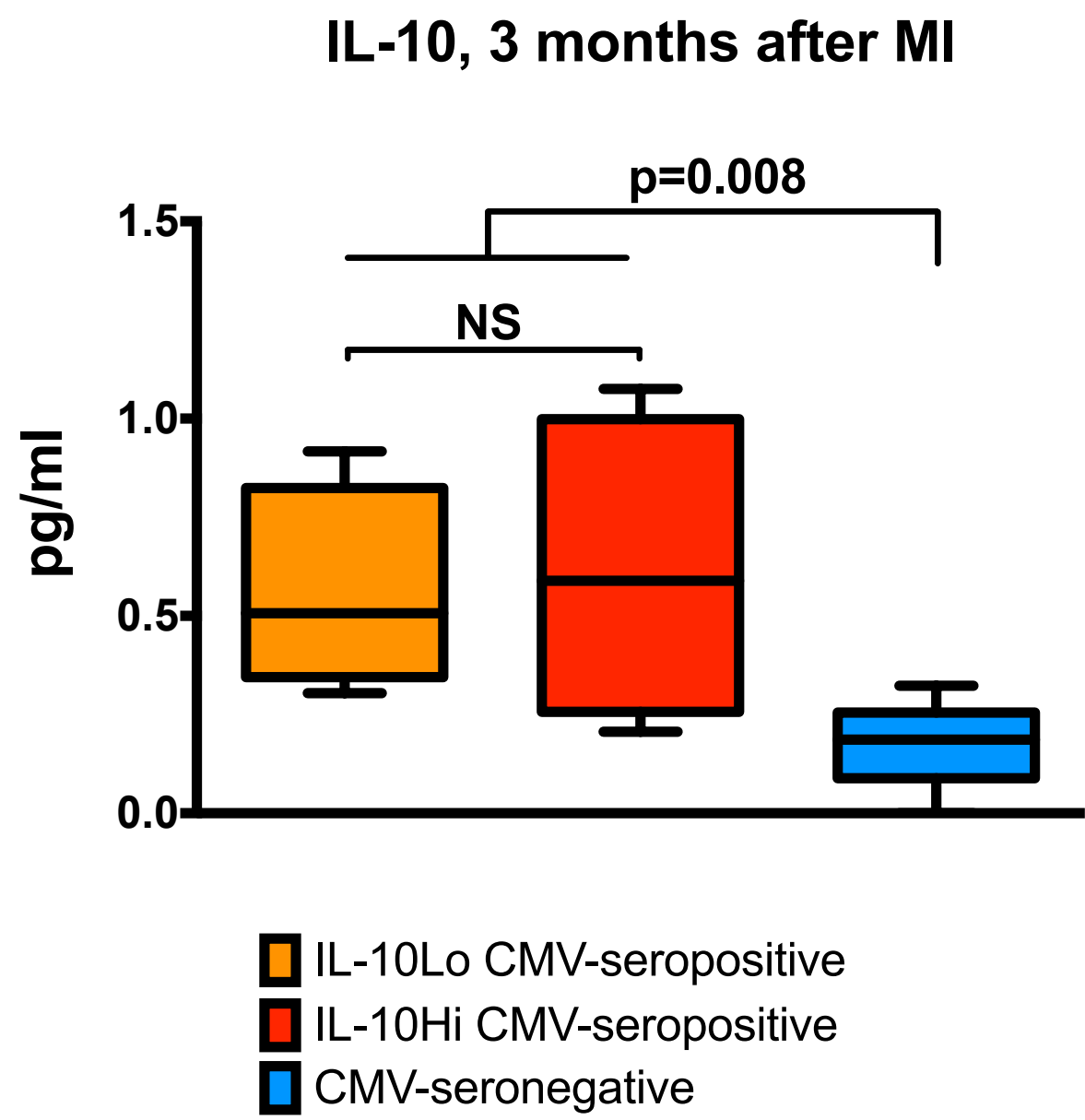

Supplement: Supplementary file 1 [file iid30003-0056-sd1.pdf]
