# Supplementary material for: Differences in immune responses between CMV-seronegative and -seropositive patients with myocardial ischemia and reperfusion
Source: Immun Inflamm Dis. 2015 Mar 1;3(2):56–70. doi: 10.1002/iid3.49 (PMC4444149; doi:10.1002/iid3.49)
Supplement: Supplementary file 2 [file iid30003-0056-sd2.docx]

| **Supplemental Table 1. Baseline characteristics of CMV-seropositive and seronegative groups of patients** | | | |
| --- | --- | --- | --- |
|  | **CMV-seropositive (n=29)** | **CMV-seronegative (n=23)** | **P value** |
| Age | 62 (54; 72) | 56 (47; 63) | 0.033 |
| Sex: male/female | 18 / 11 | 21 / 2 | 0.016 |
| EBV-seropositive | 28 (96.5%) | 18 (78.3%) | 0.040 |
| White Blood Cells, (cells/μl) | 11520 (9500; 14155) | 12540 (10938; 14903) | 0.392 |
| Lymphocytes, (cells/μl) | 2000 (1400; 3110) | 1915 (1413; 2730) | 0.669 |
| Monocytes, (cells/μl) | 800 (545; 910) | 635 (463; 833) | 0.278 |
| Neutrophils, (cells/μl) | 9220 (6800; 10615) | 9240 (7775; 10865) | 0.530 |
| Eosinophils, (cells/μl) | 100 (60; 170) | 160 (65; 310) | 0.397 |
| Platelets, (x10^3^/μl) | 239 (204; 311) | 240 (225; 326) | 0.482 |
| Serum Creatinine, (µmol/L) | 77 (65; 88) | 81 (69; 95) | 0.190 |
| HDL cholesterol, (mmol/L) | 1.2 (1.0; 1.4) | 1.2 (1.0; 1.4) | 0.795 |
| Serum cholesterol, (mmol/L) | 5.1 (4.5; 5.8) | 5.0 (4.5; 6.1) | 0.699 |
| Glucouse, (mmol/L) | 7.9 (6.1; 9.2) | 8.1 (7.3; 9.4) | 0.161 |
| Triglycerides (mmol/L) | 1.3 (0.9; 2.4) | 1.9 (1.1; 2.7) | 0.483 |
| Troponin, 12h sample (ng/L) | 4800 (2274; 8461) | 5098 (2102; 8238) | 0.934 |
| Continuous variables are represented as median (25% quartile; 75% quartile) and compared by Mann-Whitney test; categorical variables are compared by chi-square test. Listed parameters represent measurements before reperfusion, except for troponin levels (12h). | | | |

| **Supplemental Table 2. Absolute counts of lymphocyte populations in CMV-seropositive and seronegative patients at different time points** | | | | | | | | | | | | | | |
| --- | --- | --- | --- | --- | --- | --- | --- | --- | --- | --- | --- | --- | --- | --- |
| **Subpopulations** | **Time points** | | | **Median (25% quartile; 75% quartile), cells/μl** | | **P value** |  | **Subpopulations** | **Time points** | | | **Median (25% quartile; 75% quartile), cells/μl** | | **P value** |
|  |  |  |  | **CMV-seropositive patients** | **CMV-seronegative patients** |  |  |  |  |  |  | **CMV-seropositive patients** | **CMV-seronegative patients** |  |
| CD8^+^  EM T cells | Acute MI | Before PPCI | | 117 (59; 151) | 90 (49; 123) | 0.289 |  | CD4^+^  EM T cells | Acute MI | Before PPCI | | 237 (142; 355) | 179 (97; 259) | 0.099 |
|  |  | After PPCI | 15 min | 67 (46; 118) | 61 (41; 88) | 0.241 |  |  |  | After PPCI | 15 min | 181 (123; 256) | 141 (82; 226) | 0.09 |
|  |  |  | 30 min | 57 (36; 90) | 46 (29; 83) | 0.352 |  |  |  |  | 30 min | 156 (101; 213) | 130 (76; 161) | 0.2 |
|  |  |  | 90 min | 44 (33; 71) | 36 (23; 61) | 0.352 |  |  |  |  | 90 min | 142 (106; 177) | 121 (82; 175) | 0.25 |
|  |  |  | 24h | 83 (56; 129) | 85 (64; 129) | 0.94 |  |  |  |  | 24h | 246 (169; 321) | 221 (163; 297) | 0.623 |
|  | 3 months after acute MI | | | 90 (63; 134) | 91 (61; 146) | 0.975 |  |  | 3 months after acute MI | | | 237 (164; 383) | 193 (144; 272) | 0.466 |
| CD8^+^CD27^-^  EM T cells | Acute MI | Before PPCI | | 23 (10; 43) | 9 (4; 14) | **0.001*** |  | CD4^+^CD27^-^  EM T cells | Acute MI | Before PPCI | | 76 (30; 123) | 28 (18; 34) | **<0.001*** |
|  |  | After PPCI | 15 min | 15 (7; 33) | 6 (3; 10) | **0.001*** |  |  |  | After PPCI | 15 min | 51 (24; 79) | 17 (14; 25) | **<0.001*** |
|  |  |  | 30 min | 12 (6; 27) | 5 (2; 9) | **0.001*** |  |  |  |  | 30 min | 41 (20; 69) | 18 (11; 27) | **0.001*** |
|  |  |  | 90 min | 9 (5; 13) | 3 (2; 6) | **0.001*** |  |  |  |  | 90 min | 35 (18; 51) | 17 (13; 23) | **<0.001*** |
|  |  |  | 24h | 15 (7; 26) | 7 (4; 13) | **0.002*** |  |  |  |  | 24h | 64 (35; 94) | 30 (25; 46) | **0.001*** |
|  | 3 months after acute MI | | | 12 (7; 47) | 7 (3; 24) | 0.169 |  |  | 3 months after acute MI | | | 71 (35; 163) | 35 (14; 44) | **0.028*** |
| CD8^+^  TEMRA cells | Acute MI | Before PPCI | | 280 (183; 491) | 116 (71; 247) | **<0.001*** |  | CD4^+^  TEMRA cells | Acute MI | Before PPCI | | 41 (19; 87) | 21 (12; 36) | **0.014*** |
|  |  | After PPCI | 15 min | 168 (114; 301) | 65 (47; 122) | **<0.001*** |  |  |  | After PPCI | 15 min | 27 (13; 49) | 15 (9; 20) | **0.016*** |
|  |  |  | 30 min | 116 (85; 235) | 51 (39; 135) | **<0.001*** |  |  |  |  | 30 min | 26 (11; 48) | 13 (7; 20) | **0.011*** |
|  |  |  | 90 min | 114 (62; 169) | 43 (30; 70) | **<0.001*** |  |  |  |  | 90 min | 21 (10; 49) | 15 (8; 18) | **0.037*** |
|  |  |  | 24h | 261 (203; 365) | 154 (98; 224) | **<0.001*** |  |  |  |  | 24h | 41 (28; 85) | 33 (19; 42) | 0.072 |
|  | 3 months after acute MI | | | 302 (203; 427) | 157 (93; 232) | **0.013*** |  |  | 3 months after acute MI | | | 53 (22; 98) | 41 (20; 49) | 0.294 |
| CD8^+^CD27^-^  TEMRA cells | Acute MI | Before PPCI | | 208 (81; 345) | 24 (12; 49) | **<0.001*** |  | CD4^+^CD27^-^  TEMRA cells | Acute MI | Before PPCI | | 18 (4; 61) | 2 (1; 3) | **<0.001*** |
|  |  | After PPCI | 15 min | 123 (50; 233) | 16 (8; 25) | **<0.001*** |  |  |  | After PPCI | 15 min | 10 (3; 30) | 1 (1; 2) | **<0.001*** |
|  |  |  | 30 min | 77 (38; 175) | 12 (7; 17) | **<0.001*** |  |  |  |  | 30 min | 7 (2; 22) | 1 (0; 2) | **<0.001*** |
|  |  |  | 90 min | 65 (30; 110) | 9 (4; 14) | **<0.001*** |  |  |  |  | 90 min | 4 (2; 21) | 1 (1; 2) | **<0.001*** |
|  |  |  | 24h | 159 (75; 253) | 26 (12; 37) | **<0.001*** |  |  |  |  | 24h | 13 (5; 71) | 3 (2; 5) | **<0.001*** |
|  | 3 months after acute MI | | | 153 (103; 318) | 39 (14; 81) | **0.001*** |  |  | 3 months after acute MI | | | 19 (4; 86) | 1 (1; 5) | **0.007*** |
| NKT-like  cells | Acute MI | Before PPCI | | 119 (52; 319) | 37 (26; 109) | **<0.001*** |  | Treg cells | Acute MI | Before PPCI | | 9 (8;13) | 17 (15; 29) | **0.03*** |
|  |  | After PPCI | 15 min | 84 (35; 201) | 25 (16; 36) | **<0.001*** |  |  |  | After PPCI | 90 min | 9 (7; 10) | 15 (9; 20) | 0.082 |
|  |  |  | 30 min | 59 (25; 163) | 22 (15; 36) | **0.001*** |  |  |  |  | 24h | 12 (8; 18) | 17 (15; 24) | 0.125 |
|  |  |  | 90 min | 47 (25; 103) | 17 (10; 31) | **<0.001*** |  | NK cells | Acute MI | Before PPCI | | 469 (279; 750) | 419 (285; 511) | 0.861 |
|  |  |  | 24h | 100 (45; 233) | 38 (22; 72) | **0.001*** |  |  |  | After PPCI | 15 min | 267 (180; 525) | 305 (163; 414) | 0.872 |
|  | 3 months after acute MI | | | 125 (71; 192) | 34 (20; 68) | **0.002*** |  |  |  |  | 30 min | 181 (105; 344) | 222 (136; 336) | 0.762 |
|  |  | | |  |  |  |  |  |  |  | 90 min | 157 (89; 234) | 161 (125; 238) | 0.905 |
|  |  |  |  |  |  |  |  |  |  |  | 24h | 178 (130; 270) | 219 (136; 269) | 0.835 |
|  |  |  |  |  |  |  |  |  | 3 months after acute MI | | | 241 (199; 301) | 316 (230; 536) | 0.357 |
| Acute MI data from 29 CMV-seropositive and 23 CMV-seronegative patients. 3-month data from 15 CMV-seropositive and 8 CMV-seronegative individuals. P values by Mann-Whitney U test for CMV-seropositive and seronegative groups. | | | | | | | | | | | | | | |
